# Structural Uncertainty Analysis of High-Temperature Strain Gauge Based on Monte Carlo Stochastic Finite Element Method

**DOI:** 10.3390/s23208647

**Published:** 2023-10-23

**Authors:** Yazhi Zhao, Fengling Zhang, Yanting Ai, Jing Tian, Zhi Wang

**Affiliations:** Liaoning Key Laboratory of Advanced Measurement and Test Technology for Aviation Propulsion System, School of Aero-Engine of Shenyang Aerospace University, Shenyang 110136, China; zhaoyazhi@stu.sau.edu.cn (Y.Z.); ytai@163.com (Y.A.); tianjing@188.com (J.T.); wangzi629@163.com (Z.W.)

**Keywords:** high-temperature strain gauge, primary sub-model, stochastic finite element method, Monte Carlo method, uncertainty analysis

## Abstract

The high-temperature strain gauge is a sensor for strain measurement in high-temperature environments. The measurement results often have a certain divergence, so the uncertainty of the high-temperature strain gauge system is analyzed theoretically. Firstly, in the conducted research, a deterministic finite element analysis of the temperature field of the strain gauge is carried out using MATLAB software. Then, the primary sub-model method is used to model the system; an equivalent thermal load and force are loaded onto the model. The thermal response of the grid wire is calculated by the finite element method (FEM). Thermal–mechanical coupling analysis is carried out by ANSYS, and the MATLAB program is verified. Finally, the stochastic finite element method (SFEM) combined with the Monte Carlo method (MCM) is used to analyze the effects of the physical parameters, geometric parameters, and load uncertainties on the thermal response of the grid wire. The results show that the difference of temperature and strain calculated by ANSYS and MATLAB is 1.34% and 0.64%, respectively. The calculation program is accurate and effective. The primary sub-model method is suitable for the finite element modeling of strain gauge systems, and the number of elements is reduced effectively. The stochastic uncertainty analysis of the thermal response on the grid wire of a high-temperature strain gauge provides a theoretical basis for the dispersion of the measurement results of the strain gauge.

## 1. Introduction

A high-temperature strain gauge is a precision instrument for measuring the surface strain of a structure in a high-temperature environment. The high-temperature strain gauge is usually fixed on the surface of the measured structure by spraying [1]. In practical engineering, the manufacturing and installation process of the high-temperature strain gauge is often affected by a variety of uncertain factors [2]. For example, the thickness of each spray layer is often affected by technical conditions and human factors during installation. In addition, the spray material and the grid wire material physical parameters (such as density, elastic modulus, heat transfer coefficient, etc.), geometric parameters (such as length, width, and height), and external loads have uncertainty. The uncertainty of the above input conditions directly leads to the uncertainty of the output strain, making the measurement results have a certain divergence [3]. It is important for the development of high-temperature strain gauges to accurately analyze the effects of uncertainties in assembly and material parameters on the accuracy of strain gauge measurements and to determine highly sensitive uncertainty parameters.

At present, most of the research on the measurement accuracy of strain gauges are based on deterministic analysis. The influence of different geometric parameters on the accuracy is analyzed. N.M. Khairi carried out the measurements using three sizes of strain gauges (2 mm, 5 mm, and 8 mm), and the response data were compared and analyzed [4]. P. Schmid studied the effect of film thickness on the gauge factor of platinum films at different temperatures [5]. Rakesh Kolhapure used the finite element software to optimize the multi-objective geometric parameters of the sensor, which gave the optimal parameter combination. The effect of each process parameter on the performance characteristics was studied using the ANOVA technique [6]. Hao Liu studied static and dynamic strain responses from room temperature to 1073 K. The temperature dependence of the measurement factor and the periodic variation of the strain gauge resistance were analyzed [7]. Zhiyang Guo studied the performance of direct ink writing (DIW)-printed strain gauges. The results showed that their performance mainly depends on the design parameters and fabrication process [8]. Herbert Enser proposed that the difference in elastic moduli between the adhesive layer and the substrate affected the measured coefficients of the final sensor [9]. Yongqian Li concluded that the width, thickness, and shear modulus of the bond layer change greatly, and have a great influence on the strain transfer ratio. Moreover, the strain transfer ratio strongly depends on the length and width of the sensitive mesh [10]. Daniel Grabner studied the effect of temperature and strain on resistance strain gauges [11].

The uncertainty of models and parameters is a common phenomenon in engineering practice. For this reason, many scientists and engineers have studied the problem of analyzing and solving the uncertainty of models and parameters. The stochastic finite element method is formed by combining the stochastic method with the finite element method. Mikkel Løvenskjold Larse established stochastic stiffness matrices and stress stiffness matrices for stochastic and buckling analysis in beam and frame problems. Furthermore, the parameters were discretized by Karhunen–Loève and solved [12]. Bartłomiej Pokusinski presented a selection procedure for a random perturbation method. The convergence and accuracy of generalized perturbation schemes in linear and nonlinear problems of solid mechanics were numerically analyzed [13]. Chen Chen used the stochastic Galerkin method to realize the discretization of the physical space and the probability space to advance the solution [14]. Jian Li used the stochastic Navier–Stokes equations, which gave the corresponding results for the fully discrete finite element method [15].

In 1972, Shinozuka first applied the Monte Carlo method to the field of engineering mechanics. In 1976, he converted the two-dimensional random plate problem into a format compatible with the finite element [16]. In 2005, Popescu used a Monte Carlo simulation (MCS) and finite element analysis (FEA) combined method to predict random shear strength to study the influence of the random heterogeneity of soil properties and the behavior of failure mechanism [17]. In 2006, MCS stochastic finite element analysis was used to optimize the reliability design of the shell structure, considering the material defects and uncertain thickness [18]. The Monte Carlo method for the finite element model has a good applicability; the calculation results are accurate, but needs to consume a large amount of computer resources. This difficulty with the development of computer technology and the finite element method, to a certain extent, has been solved. The Monte Carlo stochastic finite element method has been widely used. In 2019, Lorella Palluotto simulated the radiative heat transfer of three actual three-dimensional configurations based on the Monte Carlo method. And a stochastic quasi-Monte-Carlo algorithm, which was superior to Monte Carlo algorithm, was developed [19]. In 2020, Shashank Vadlamani proposed the stochastic beam element formulation. The results obtained by the perturbation method were compared with those obtained by the MCS [20]. In 2020, Bing Wang analyzed the uncertainty and reliability of thermomechanical problems through MCS. The accuracy and effectiveness of the smooth finite element method based on randomly stable nodes were verified [21]. In 2023, in Ngoc-Tu Do’s study based on the stochastic finite element method of MCS, the stochastic vibration of functionally graded material (FGM) plates under moving loads was studied when the parameter inputs were random quantities following normal distribution [22].

In summary, nowadays, the research on high-temperature strain gauges is mainly divided into two main areas: experiment and simulation. High-temperature strain gauges are expensive and it is difficult to obtain sufficient data in tests. The simulation can only perform deterministic calculations and cannot analyze uncertain parameters. Therefore, a high-temperature strain gauge simulation model capable of uncertainty parameter analysis is developed by using MATLAB programming in this paper. The accuracy of the calculation results of the MATLAB program is then verified by Workbench simulation results. Finally, the thermal response of high-temperature strain gauges is analyzed by combining the Monte Carlo stochastic finite element method and the random factor method.

## 2. Analysis of Temperature Field of High-Temperature Strain Gauge

### 2.1. Problem Description

**Figure 1:**
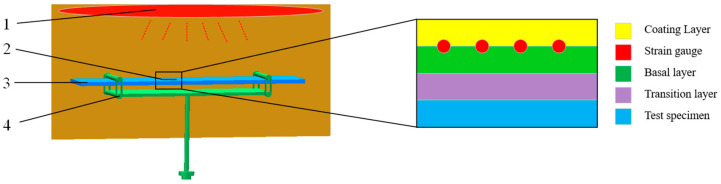
Strain gauge installation and load. 1—heating device; 2—strain gauge; 3—specimen; 4—the loading device of the force.

It is of little significance to study the performance of a single and free strain gauge, which needs to be installed on the specimen for analysis. As shown in Figure 1, the transition layer is first sprayed on the specimen, followed by the basal layer. The strain gauge is placed on the basal layer, and finally the covering layer is sprayed to fix it. The strain gauge, basal layer, and coating layer form the complete measurement system, and the function of the transition layer is to connect the measurement system to the test specimen. In order to better simulate the high-temperature environment of the engine, the specimen installed with the strain gauge is placed in the high-temperature heating furnace. The temperature and force are loaded together.

### 2.2. Establishment of the Primary Sub-Model

In the high-temperature strain gauge structure, the thickness of the specimen is 6 mm, the thickness of the transition layer is 0.1 mm, and the thickness of the basal layer is 0.1 mm. The cross-section of the grid wire of the strain gauge is circular, the diameter is 0.02 mm, and the thickness of the cover layer is 0.3 mm. In the transverse direction, the heat is uniform, in the longitudinal direction due to the fact that the size difference is large. The method of step-by-step fine analysis, that is, the primary sub-model method, is used for grid division, reducing the requirements for computing resources.

**Figure 2:**
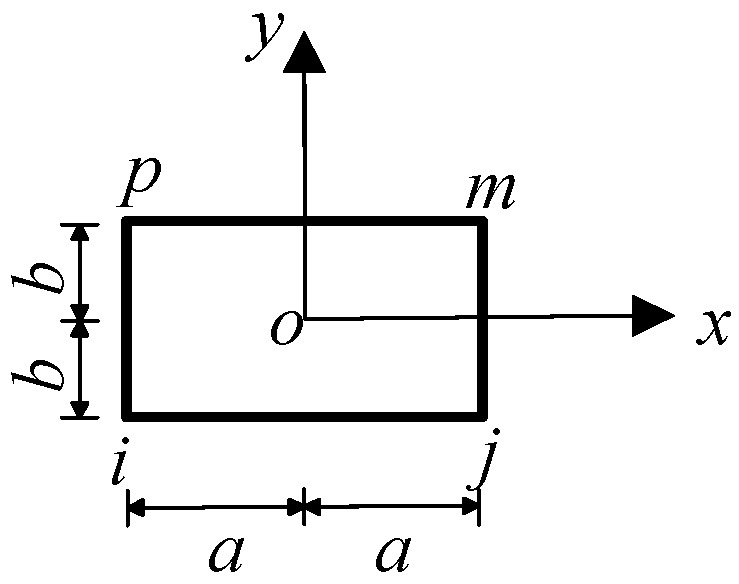
Planar four-node rectangular element.

Compared with the three-node triangular element, the calculation accuracy of the planar four-node rectangular element is higher. Therefore, the primary sub-model adopts the planar four-node rectangular element for grid division, as shown in Figure 2. The establishment of the primary sub-model is mainly divided into two steps. The first step is to establish the entire longitudinal model from the test specimen to the cover layer. The large grid division is used for the preliminary calculation. The side length of the element is 2*a =* 2*b =* 0.1 mm. In the second step, the basal and cover layer, which contact the grid wire, are cut out from the whole, and the local area is divided into fine grids. The element is a square with a side length of 2*a* = 2*b* = 0.01 mm, and the temperature boundary conditions calculated by the first step are applied to the boundary for a more detailed calculation.

**Figure 3:**
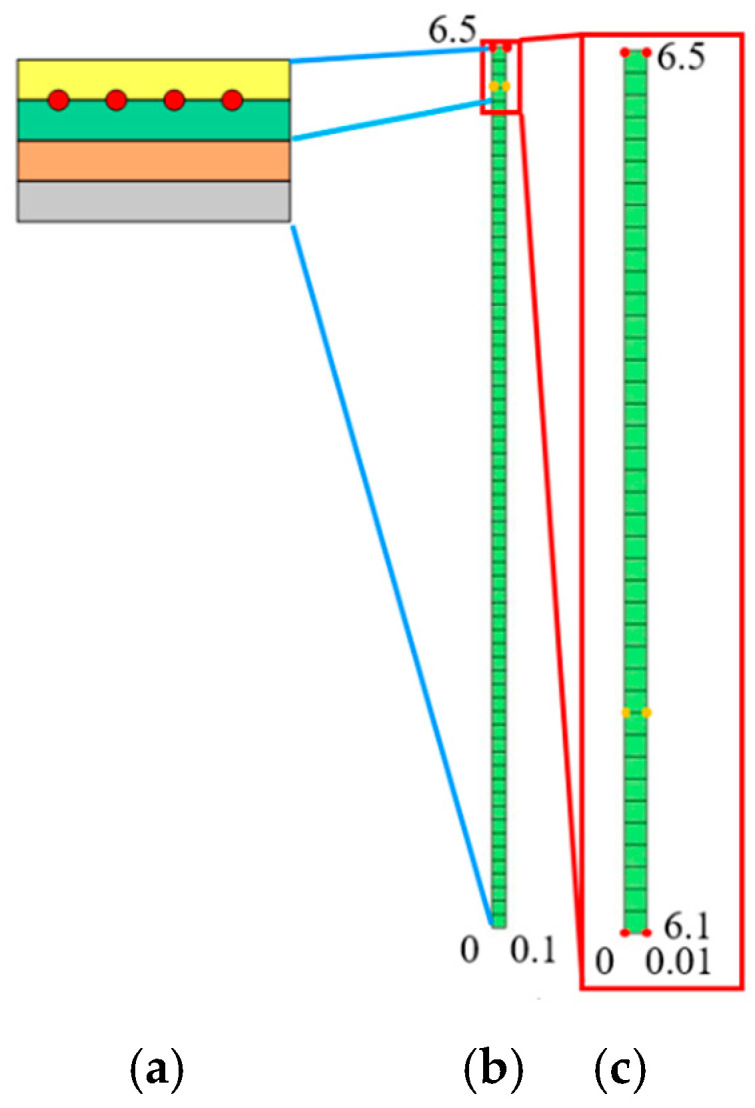
Primary sub-model. (**a**) diagram of each bond layer; **(b**) primary model; (**c**) sub model.

The grid division of the primary sub-model is created using a MATLAB program [23]. Figure 3a is the diagram of each bond layer, the primary model is shown in Figure 3b, and Figure 3c shows the sub model. The boundary condition in the main model is the temperature load applied to the upper surface of 1273.15 K. The node temperature calculated by the primary model is loaded into the sub-model as the boundary condition, and the loading node is represented by red dots as shown in Figure 3.

### 2.3. Finite Element Calculation of Temperature Field

The governing equation of the thermal transfer problem based on the Fourier heat transfer law and the energy conservation law is as follows:

(1) $$\frac{\partial }{\partial x}(k_{x}\frac{\partial T}{\partial x})+\frac{\partial }{\partial y}(k_{y}\frac{\partial T}{\partial y})=0$$

where *k_x_* and *k_y_* are the thermal conduction coefficients along the *x* and *y* directions, respectively, and the unit is W/(m·K). On the basis of Equation (1), the integral formula of the element thermal transfer matrix $K_{T}^{e}$ can be obtained as Equation (2):

(2) $$K_{T}^{e}=\iint \left[k_{x}\frac{\partial {\left[N\right]}^{T}}{\partial x}\cdot \frac{\partial \left[N\right]}{\partial x}+k_{y}\frac{\partial {\left[N\right]}^{T}}{\partial x}\cdot \frac{\partial \left[N\right]}{\partial x}\right]dxdy+\int_{S_{3}^{e}}\bar{h_{c}}N^{T}NdA$$

The internal element heat transfer matrix of the specimen in the primary model is achieved as follows:

$$K_{T1}^{e}=\left[\begin{array}{cccc} 13.33 & -3.33 & -3.33 & -6.67\\ -3.33 & 13.33 & -6.67 & -3.33\\ -3.33 & -6.67 & 13.33 & -3.33\\ -6.67 & -3.33 & -3.33 & 13.33 \end{array}\right]$$

The internal element heat transfer matrix of the transition layer is obtained as:

$$K_{T2}^{e}=\left[\begin{array}{cccc} 16.67 & -4.17 & -4.17 & -8.33\\ -4.17 & 16.67 & -8.33 & -4.17\\ -4.17 & -8.33 & 16.67 & -4.17\\ -8.33 & -4.17 & -4.17 & 16.67 \end{array}\right].$$

The internal element thermal transfer matrix of overlay layer can be described as:

$$K_{T3}^{e}=\left[\begin{array}{cccc} 22 & -5.5 & -5.5 & -11\\ -5.5 & 22 & -11 & -5.5\\ -5.5 & -11 & 22 & -5.5\\ -11 & -5.5 & -5.5 & 22 \end{array}\right].$$

From Formula (2), notice that the heat transfer coefficients of different bonding layers are different [24], resulting in different element heat transfer matrices. The element heat transfer matrix of each layer is calculated by MATLAB program. The element heat transfer matrix of the same bonding layer is assembled first. Then, the thermal transfer matrix of different bonding layers is assembled. Finally, the global heat transfer matrix $K_{T}^{e}$ is obtained.

For the primary model, the temperature of the upper boundary is loaded at 1273 K. The temperature is loaded on the two nodes marked in red, as shown in Figure 3b. The value of temperature assigned to the element node is calculated by the formula as $P_{T}^{e}=\int_{S_{3}}\bar{h_{c}}T_{\infty }\cdot NTd\Omega ={\left[\begin{array}{cccc} 0 & 0 & 50 & 50 \end{array}\right]}^{T}$; the internal element has no thermal load. The node temperature array of the boundary element and the internal element are assembled into the global temperature array ***P****_T_*.

The global heat transfer matrix $K_{T}$ and load array ***P****_T_* were substituted into the global equilibrium Equation (3) to solve the problem.

(3) $$K_{T}\cdot q_{T}=P_{T}$$

The temperature values of each node in the primary model are obtained, and the calculation results of some nodes are shown in Table 1. The *y* co-ordinate of the cutting position of the primary sub-model is 6.1 mm, that is, the positions of nodes No. 62 and 128.

**Figure 4:**
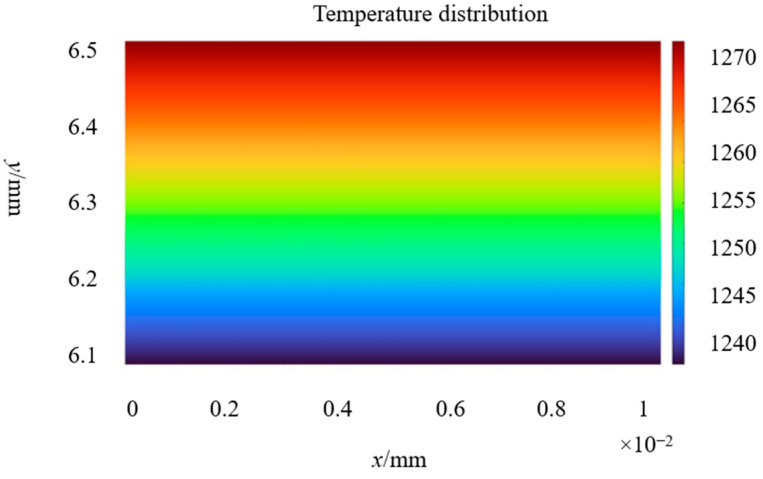
Temperature distribution cloud of the sub-model.

The temperature of nodes No. 62 and 128 is applied to the lower boundary of the sub-model as a load, and the upper boundary of the sub-model is also subjected to a temperature load of 1273 K, as shown by the red dot in Figure 3c. The temperature profile calculated of the sub-model drawn by MATLAB is shown in Figure 4. The specific values of the temperature of each node are shown in Table 2. The *y* co-ordinate of the position of the grid wire is 6.2 mm, which is the position of nodes 11 and 52.

## 3. Thermal Response Analysis of High-Temperature Strain Gauge Based on Coupled Thermo-Mechanical Model

### 3.1. Establishment of a Primary Sub Model for Thermal Response Analysis

On the basis of a temperature field analysis, the FEM is used to further analyze the thermal response of the high-temperature strain gauge [25]. As shown in Figure 1, the force is loaded on the specimen and transferred to the grid wire of the strain gauge through the bonding layers, rather than directly applying the force load to the strain gauge. The size difference between the specimen and the strain gauge is very large. The two-dimensional size of the specimen is 400 mm × 6 mm, and the size of the strain gauge is 7.8 mm × 1.2 mm. The load of the strain gauge comes from the specimen and the specimen is almost unaffected by the strain gauge. Based on the above-mentioned reasons, the primary sub-model is used to analyze the thermal response of the strain gauge. It can effectively reduce the number of elements and obtain the exact thermal response of the concerned grid wire part.

**Figure 5:**
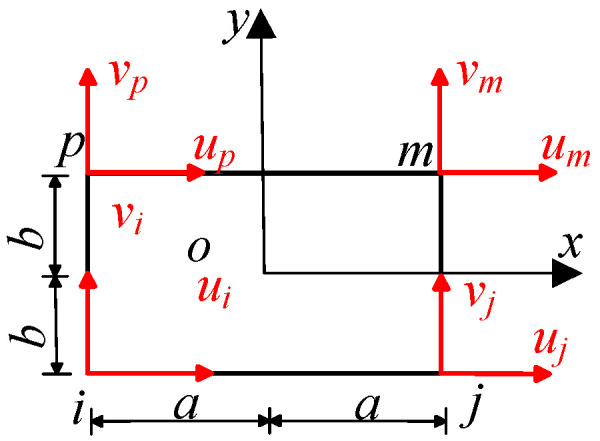
Planar four-node eight-degree-of-freedom rectangular unit.

**Figure 6:**
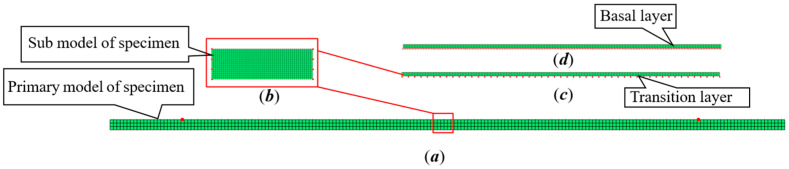
Grid division of the primary sub-model. (**a**) primary model of specimen; (**b**) sub model of specimen; (**c**) transition layer; (**d**) basal layer.

A rectangular element with four nodes and eight degrees of freedom are used to divide the grid, as shown in Figure 5. The element of the primary model is a square with a side length of 2*a =* 2*b* = 2 mm, with a total of 3 × 200 elements. In order to facilitate the uncertainty analysis of the thermal response and to facilitate the MATLAB program, the assembly of the stiffness matrix between different bonding layers is omitted, and the sub-model is divided into three parts. In this way, in the following uncertainty analysis, when a certain parameter is assumed to be a random variable, the whole stiffness matrix need not be assembled repeatedly, which is very important when the number of samples is large. The purpose of this is to effectively reduce the amount of computation. This practice refers to the transfer of force between layers in the theoretical calculation [10,26]. The load force is loaded on the specimen and transferred to the grid wire of the strain gauge through the transition layer and the substrate. The sub-model is divided into three parts, namely, sub-models 1, 2 and 3, as shown in Figure 6b–d.

The square element side length of sub-model 1 is 2*a* = 2*b* = 0.4 mm, with a total of 15 × 50 units. The square unit side length of sub-model 2 and 3 is 2*a* = 2*b* = 0.1 mm, with 1 × 200 elements, respectively. The grid division of the primary sub-model is realized by a MATLAB program, and the results are shown in Figure 6.

### 3.2. Transfer of Force in the System

The shape function matrix ***N***_1_ of the rectangular element of the primary model is:

(4) $$\begin{array}{cc} N_{1} & =\left[\begin{array}{cccc} N_{i} & N_{j} & N_{m} & N_{p} \end{array}\right]\\ & =\frac{1}{4}[\begin{array}{cccc} \left(1-x)(1-y\right) & \left(1+x)(1-y\right) & \left(1+x)(1+y\right) & \left(1-x)(1+y\right) \end{array}] \end{array}$$

The strain-displacement matrix ***B***_1_ of the primary model element is:

$$B_{1}(x,y)=\left[\begin{array}{cc} \frac{\partial }{\partial x} & 0\\ 0 & \frac{\partial }{\partial y}\\ \frac{\partial }{\partial y} & \frac{\partial }{\partial x} \end{array}\right]N_{1}=\left[\begin{array}{cccccccc} \frac{y-1}{4} & 0 & \frac{1-y}{4} & 0 & \frac{y+1}{4} & 0 & \frac{-1-y}{4} & 0\\ 0 & \frac{x-1}{4} & 0 & \frac{-x-1}{4} & 0 & \frac{1+x}{4} & 0 & \frac{1-x}{4}\\ \frac{x-1}{4} & \frac{y-1}{4} & \frac{-x-1}{4} & \frac{1-y}{4} & \frac{1+x}{4} & \frac{1+y}{4} & \frac{1-x}{4} & \frac{-1-y}{4} \end{array}\right]$$

The specimen is made of Ni-Al superalloy GH36, which is commonly used in hot end parts of aircraft engines. The elastic modulus of the specimen *E*_1_ = 1.33 × 10^11^ Pa, Poisson’s ratio *μ*_1_ = 0.4, and the elastic coefficient matrix ***D*** are obtained by Equation (5).

(5) $$D=\frac{E}{1-\mu^{2}}\left[\begin{array}{ccc} 1 & \mu & 0\\ \mu & 1 & 0\\ 0 & 0 & \frac{1-\mu }{2} \end{array}\right]=1.58\times 10^{11}\left[\begin{array}{ccc} 1 & 0.4 & 0\\ 0.4 & 1 & 0\\ 0 & 0 & 0.3 \end{array}\right]$$

By substituting ***B***_1_ and ***D*** into Equation (5), the element stiffness matrix $K_{1}^{e}$ of the specimen is:

(6) $$\begin{array}{l} K_{1}^{e}=\int_{\Omega^{e}}B_{1}^{T}DB_{1}d\Omega \\ =10^{6}\times \left[\begin{array}{cccccccc} 6.86 & 2.77 & -4.49 & 3.96 & -3.43 & -2.77 & 1.06 & -0.4\\ 2.77 & 6.86 & -0.4 & 1.06 & -2.77 & -3.43 & 3.96 & -4.49\\ -4.49 & -0.4 & 6.86 & -2.77 & 1.06 & 3.96 & -3.43 & 2.77\\ 0.4 & 1.06 & -2.77 & 6.86 & -0.4 & -4.49 & 2.77 & -3.43\\ -3.43 & -2.77 & 1.06 & -0.4 & 6.86 & 2.77 & -4.49 & 3.96\\ -2.77 & -3.43 & 3.96 & -4.49 & 2.77 & 6.86 & -0.4 & 1.06\\ 1.06 & 0.4 & -3.43 & 2.77 & -4.49 & -0.4 & 6.86 & -2.77\\ -3.96 & -4.49 & 2.77 & -3.43 & 3.96 & 1.06 & -2.77 & 6.86 \end{array}\right] \end{array}$$

The element stiffness matrix is assembled to form the global stiffness matrix ***K***, and ***q*** is the node displacement array. The total load is superimposed by nodal force vector ***P***_1_ and equivalent temperature load ***P***_01_. The element equivalent temperature load $P_{01}^{e}$ is related to temperature increment Δ*T* and thermal expansion coefficient *α*_1_, as shown in Equation (7), Δ*T* = 1223.15 K, *α*_1_ = 17 × 10^−6^/K.

(7) $$P_{0}{}^{e}={\int_{\Omega^{e}}B_{\_1}}^{T}D\varepsilon^{0}d\Omega$$

(8) $$\varepsilon^{0}=\alpha_{1}\Delta T{\left[\begin{array}{ccc} 1 & 1 & 0 \end{array}\right]}^{T}=10^{-2}\times {\left[1.615\;1.615\;0\right]}^{T}$$

Substitute ***K***, ***P***_1_, and ***P***_01_ into the global stiffness Equation (9) and solve it. The displacements ***q*** of all nodes divided in the primary model are obtained:

(9) $$Kq=P_{1}+P_{01}$$

The concerned part, that is, the part with the strain gauge installed, is cut from top to bottom. The cutting process includes the boundary load, and a sub-model is established, as shown in Figure 6. The sub-model includes the part cut from the specimen, the transition layer, and the base, and is calculated step by step.

The node displacement ***q*** calculated by Equation (9) is substituted into Equation (10). The element on the cutting boundary of the primary model is calculated. The nodal force $P^{e}$ of the boundary element is obtained. The nodal force is taken as the external load and loaded into sub-model 1, as shown by the red dot in Figure 6b.

(10) $$K^{e}q^{e}=P^{e}$$

Sub-models 1, 2, and 3 are calculated by MATLAB programs similar to the primary model. The node displacements and the node forces of the boundary elements are obtained. The obtained nodal forces are transferred as loads from the specimen to the transition layer, and then to the substrate. Finally, the nodal forces of the substrate elements in contact with the strain gauge grid wire are obtained.

### 3.3. Element Division and Thermal Response Analysis of Grid Wire

**Figure 7:**
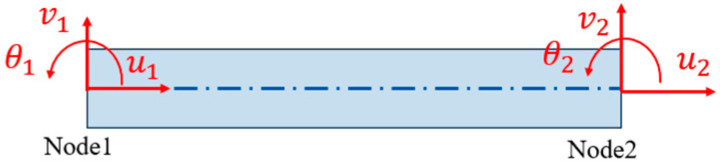
Plane beam element.

**Figure 8:**
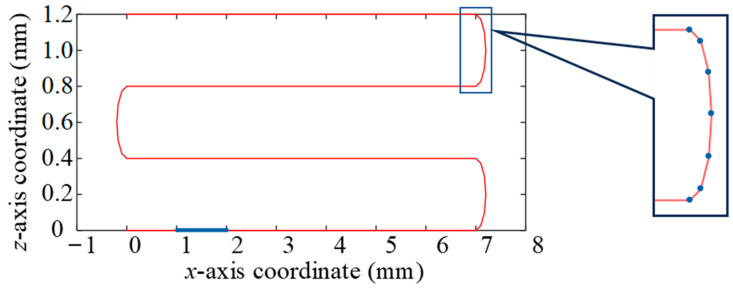
Grid wire unit division.

The division element of the strain gauge is a general beam element with two nodes and six freedom degrees, as shown in Figure 7. The element size of the linear part of the grid wire is *l*_1_ = 1 mm. The curved part is a semicircle of *r* = 0.4 mm, which is divided by angle and divided into one element every 30°. The element length $l_{2}=0.8\times \sin(\frac{\pi }{12})$ mm. The discrete result of the grid wire is shown in Figure 8.

The shape function matrix ***N*_2_** of the beam element is:

(11) $$N_{2}=\left[1-x\;1-3x^{2}+2x^{3}\;x-2x^{2}+x^{3}\;x\;3x^{2}-2x^{3}\;x^{3}-x^{2}\right]$$

where *x* represents the distance from a point to a node in the beam element.

The strain-displacement matrix ***B***_2_ is:

(12) $$B_{2}=\left[-1\;-\hat{y}(12x-6)\;-\hat{y}(6x-4)\;1\;\hat{y}(12x-6)\;-\hat{y}(6x-2)\right]$$

where $\hat{y}$ is the distance from the point on the cross-section to the neutral layer.

Take the elastic modulus of grid wire *E*_4_ = 2.2 × 10^11^ Pa, cross-section area $A_{4}=\pi r^{2}=\pi \times 0.01^{2}{\;\mathrm{mm}}^{2}$, and moment of inertia $I=\frac{\pi d^{4}}{64}=7.85\times 10^{-9}{\;\mathrm{mm}}^{4}$. In the local co-ordinate system, the stiffness matrix of a general planar beam element $K_{4}^{e}$ is calculated by Equation (13).

(13) $$K_{4}^{e}=\left[\begin{array}{cccccc} \frac{EA}{l} & 0 & 0 & -\frac{EA}{l} & 0 & 0\\ 0 & \frac{12EI}{l^{3}} & \frac{6EI}{l^{2}} & 0 & -\frac{12EI}{l^{3}} & \frac{6EI}{l^{2}}\\ 0 & \frac{6EI}{l^{2}} & \frac{4EI}{l} & 0 & -\frac{6EI}{l^{2}} & \frac{2EI}{l}\\ -\frac{EA}{l} & 0 & 0 & \frac{EA}{l} & 0 & 0\\ 0 & -\frac{12EI}{l^{3}} & -\frac{6EI}{l^{2}} & 0 & \frac{12EI}{l^{3}} & -\frac{6EI}{l^{2}}\\ 0 & \frac{6EI}{l^{2}} & \frac{2EI}{l} & 0 & -\frac{6EI}{l^{2}} & \frac{4EI}{l} \end{array}\right]$$

Through Equation (13), the stiffness matrix of the linear part of the grid wire $K_{41}^{e}$ can be obtained.

$$K_{41}^{e}=\left[\begin{array}{cccccc} 69115 & 0 & 0 & -69115 & 0 & 0\\ 0 & 20.7 & 0.01 & 0 & -20.7 & 0.01\\ 0 & 0.01 & 6.9\times 10^{-6} & 0 & -0.01 & 3.45\times 10^{-6}\\ -69115 & 0 & 0 & 69115 & 0 & 0\\ 0 & -20.7 & -0.01 & 0 & 20.7 & -0.01\\ 0 & 0.01 & 3.45\times 10^{-6} & 0 & -0.01 & 6.9\times 10^{-6} \end{array}\right]$$

The stiffness matrix of the curved part of the grid wire is $K_{42}^{e}$:

$$K_{42}^{e}=\left[\begin{array}{cccccc} 667600 & 0 & 0 & -667600 & 0 & 0\\ 0 & 18653 & 0.97 & 0 & -18653 & 0.97\\ 0 & 0.97 & 6.66\times 10^{-5} & 0 & -0.97 & 3.33\times 10^{-5}\\ -667600 & 0 & 0 & 667600 & 0 & 0\\ 0 & -18653 & -0.97 & 0 & 18653 & -0.97\\ 0 & 0.97 & 3.33\times 10^{-5} & 0 & -0.97 & 6.66\times 10^{-5} \end{array}\right]$$

Since the local co-ordinate system of the bending part does not coincide with the global co-ordinate system, as shown in Figure 8, the stiffness matrix needs to be co-ordinate-transformed through Equation (14). In Equation (15), the angle *α* between each beam element and the horizontal direction is *α*_1_ = 15°; *α*_2_ = 45°; *α*_3_ = 75°; *α*_4_ = 105°; *α*_5_ = 135°; and *α*_6_ = 165°.

(14) $$\bar{K_{42}^{e}}=T^{eT}\cdot K^{e}\cdot T^{e}$$

where

(15) $$T^{e}=\left[\begin{array}{cccccc} \cos\alpha & \sin\alpha & 0 & 0 & 0 & 0\\ -\sin\alpha & \cos\alpha & 0 & 0 & 0 & 0\\ 0 & 0 & 1 & 0 & 0 & 0\\ 0 & 0 & 0 & \cos\alpha & \sin\alpha & 0\\ 0 & 0 & 0 & -\sin\alpha & \cos\alpha & 0\\ 0 & 0 & 0 & 0 & 0 & 1 \end{array}\right]$$

The stiffness matrix of all elements is assembled to obtain the global stiffness matrix ***K***_4_, the dimensions of which is 138 × 138.

The node displacement matrix of the beam element is $q^{e}$, each node has three degrees of freedom, and the whole node displacement matrix ***q***_2_ is formed after assembly.

(16) $$q^{e}={\left[\begin{array}{cccccc} u_{1} & v_{1} & \theta_{1} & u_{2} & v_{2} & \theta_{2} \end{array}\right]}^{T}$$

The boundary condition ***P*** is the superposition of the force ***P***_2_ and the temperature equivalent load ***P***_02_. ***P***_2_ is the node force in contact with the grid wire on the basal layer calculated in Section 4.2. $P_{02}^{e}$ is the equivalent temperature load of the element which is expressed in Equation (17), taking Δ*T*_2_ = 1223.15 K, *α*_2_ = 9. 5 × 10^−6^/K, and Poisson ratio *μ* = 0.36.

(17) $$P_{02}^{e}=\int_{\Omega^{e}}B_{2}{}^{T}D_{2}\varepsilon_{2}^{0}d\Omega$$

Of which

(18) $$D_{2}=2.53\times 10^{11}\left[\begin{array}{ccc} 1 & 0.36 & 0\\ 0.36 & 1 & 0\\ 0 & 0 & 0.32 \end{array}\right]$$

(19) $$\varepsilon_{2}^{0}=\alpha_{2}\Delta T_{\_2}{\left[\begin{array}{ccc} 1 & 1 & 0 \end{array}\right]}^{T}=10^{-3}\times {\left[9.025\;9.025\;0\right]}^{T}$$

By substituting ***K***, ***P***_2_, and ***P***_02_ into the global stiffness Equation (20), the node displacement matrix ***q***_2_ is obtained.

(20) $$Kq_{2}=P_{2}+P_{02}$$

Calculate the stress and strain of one element on the grid wire. For example, take element 2, as shown in the blue line segment in Figure 8; read the displacement of node 2 and node 3 as calculated by Equation (20). Moreover, the node displacement $q^{e}$ and the geometric function matrix B(ξ) are substituted into Equations (21) and (22) to calculate the stress ***σ*** and strain *ε* of element 2. The strain field is expressed in Equation (21).

(21) $$\varepsilon (x,\hat{y})=B(\xi )\cdot q^{e}$$

The stress field is defined in Equation (22):

(22) $$\sigma (x,\hat{y})=E\cdot B(x,\hat{y})\cdot q^{e}$$

Finally, the strain of element 2 is 9200 *με,* and the *x*-direction normal stress of node 2 $\sigma_{xx}=-0.6370$ Pa, while the *y*-direction normal stress $\sigma_{yy}=1.8386$ Pa.

## 4. Results and Discussion

### 4.1. ANSYS Verification of the MATLAB Program

**Figure 9:**
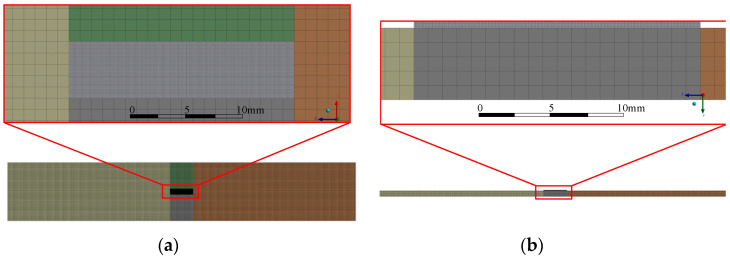
Primary model—three-dimensional finite element model of specimen and bond layer: (**a**) top view; and (**b**) front view.

**Figure 10:**
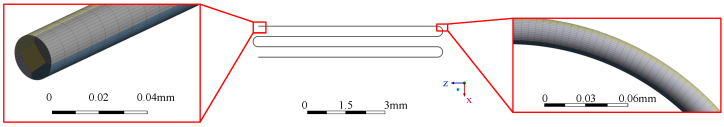
Sub-model—three-dimensional finite element model of grid wire of strain gauge.

In order to verify the correctness of the MATLAB program, a thermal–mechanical coupling simulation of the high-temperature strain gauge is carried out by ANSYS. SolidWorks is used to establish the strain gauge system model. The model is divided into regions and imported into Workbench to facilitate load application and grid division. The grid division result of the primary model is shown in Figure 9. The specimen element size is 1 mm × 1 mm × 1 mm, and each bonding layer element is 0.1 mm × 0.1 mm × 0.1 mm. The sub-model is the grid wire of the strain gauge, and its cross-section is circular. The grid wire is divided into five regions and divided by sweeping mesh. The solid model of the grid wire is obtained, as shown in Figure 10.

**Figure 11:**
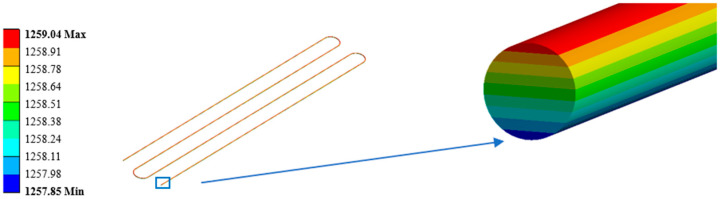
Strain gauge grid wire temperature contour.

**Figure 12:**
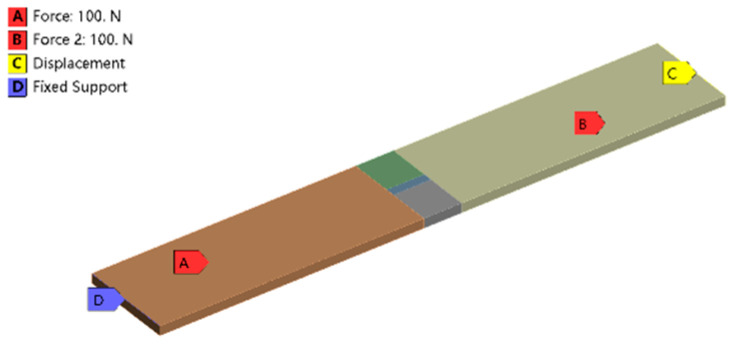
Boundary conditions and forces.

**Figure 13:**
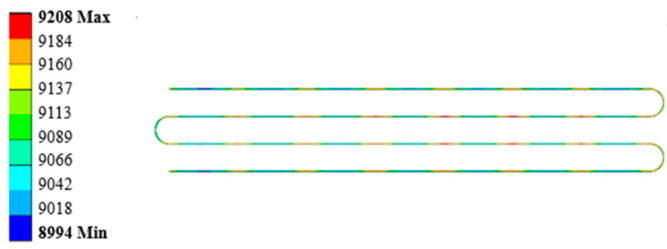
Thermal strain of strain gauge grid wire.

When a thermal load of 1273.15 K is applied to the covering layer, the temperature nephogram of the strain gauge grid wire is obtained, as shown in Figure 11. The mean temperature of the strain gauge calculated by ANSYS is 1258.44 K, compared with the temperature of 1245.2 K calculated by MATLAB; the difference between the two is 1.34%. The boundary conditions of the constraints and forces applied to the specimen are shown in Figure 12. The thermal response is calculated by superimposing the thermal load; the results are shown in Figure 13. The thermal strain at element 2 is 9141 με, compared with the equivalent thermal strain 9200 με calculated by MATLAB; the difference between the two is 0.64%. Because the difference between the calculation results of the temperature and thermal strain is small, it is considered that the modeling and calculation by the MATLAB program are correct and effective.

### 4.2. Uncertainty Analysis of Thermal Response of High-Temperature Grid Wire Based on SFEM

Under the combined action of thermal energy and force, the stress and strain finally transferred to the grid wire of the strain gauge are affected by a variety of factors. The uncertain factors include three aspects: physical parameters, geometric size, and loads, as shown in Table 3 [27,28]. The uncertainty of these factors directly leads to the uncertainty of the thermal response of the grid wire.

The normrnd generator in the MATLAB software is used to generate random numbers that obey Gaussian distribution. The program segment $r=a+{\left(b-a\right)}_{\cdot }*normrnd(M,1);$ is used to generate *M* random numbers in the interval (*a*, *b*). In order to draw a sufficient number of samples, *M* = 10^4^ is taken.

**Figure 14:**
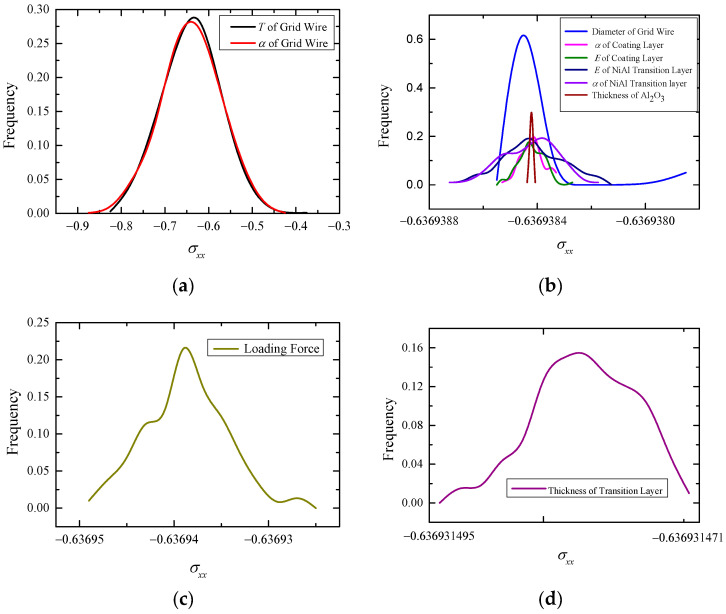
The influence of each factor uncertainty on *σ_xx_*: (**a**) *T* and *α*_4_; (**b**) *d*_4_, *α*_3_, *E*_3_, *α*_2_, *E*_2_, and *h_3_*; (**c**) F; and (**d**) *h*_2_.

Using the SFEM combined with the MCM, the variable is assumed to be a stochastic variable with a Gaussian distribution [29]. For example, set $E=normrnd(\bar{X_{E}},S_{E},\left[1,n\right])$ by MATLAB, where $\bar{X_{E}}$ is the mean value of the elastic modulus, and $S_{E}$ is the standard deviation, $S_{E}=\bar{X_{E}}\times cov$. Taking *cov* = 0.1 for each factor, the stochastic thermal response *σ_xx_* probability density curves of the high-temperature strain gauge for the above 10 uncertain factors are obtained by the stochastic finite element method (SFEM), as shown in Figure 14.

As can be seen from Figure 14, the probability density distribution of the thermal response basically follows the Gaussian distribution. Through comparison, it can be found that, among the 10 uncertain factors, the uncertainty of the thermal expansion coefficient of the grid wire α_4_ and the temperature of the grid wire *T*_4_ have the greatest influence on the dispersion of *σ_xx_*. It can be seen in Figure 14a that, when *α*_4_ follows the normal distribution (9.5 × 10^−6^, 0.95 × 10^−6^), the mean value of thermal stress *σ_xx_* of the grid wire is −0.6394 Pa, and the variance is 0.06404 Pa. When *T*_4_ obeys the normal distribution (970, 97), the mean value of thermal stress *σ_xx_* of the grid wire is −0.6387 Pa, and the variance is 0.06211 Pa. The variance of the thermal response corresponding to other factors is below 4 × 10^−6^, as shown in Figure 14b–d. Therefore, the thermal expansion coefficient of the grid wire *α*_4_ and the temperature of the grid wire *T*_4_ are determined as the primary sources of uncertainty.

### 4.3. Uncertainty Analysis of Thermal Expansion Coefficient of Grid Wire Based on SFEM

**Figure 15:**
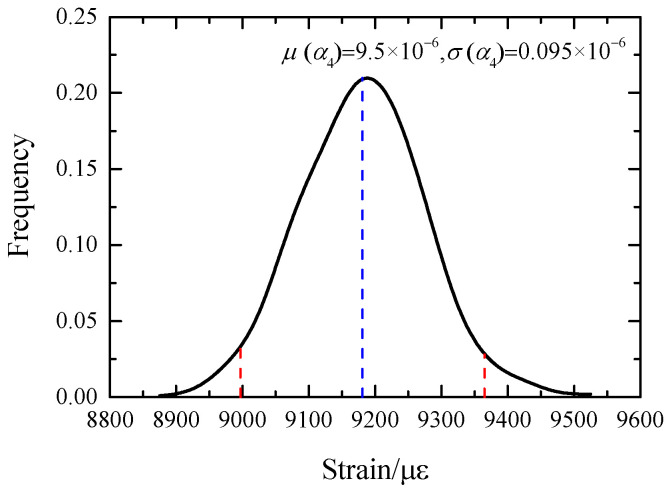
Probability density curve of element 2 thermal strain.

When the input quantity, the thermal expansion coefficient of the grid wire *α*_4_, follows the normal distribution (9.55 × 10^−6^, 0.0955 × 10^−6^), the probability density curve of the output grid wire thermal strain *ε* follows the normal distribution, basically, as shown in Figure 15, with the mean value of 9180.7 με and the variance of 92.05 με. The calculated 95% confidence interval is [8996.64, 9364.86].

**Figure 16:**
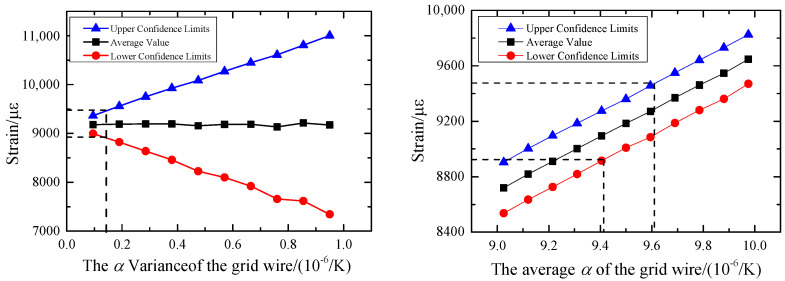
Influence of digital characteristics of *α*_4_ on 95% confidence intervals for thermal strain *ε*.

When the mean value of *α*_4_ is unchanged, its variance is changed. The coefficient of variation *cov* is uniformly selected with 10 values between [0.01, 0.1], and the probability density curve corresponding to the output thermal strain *ε* is calculated, respectively. On this basis, 95% confidence intervals under each variance are calculated, as shown in Figure 16. The confidence interval of the output *ε* increases with the increase of the variance of the thermal expansion system *α*_4_. The overall fluctuation of the mean is small, but the amplitude of the fluctuation also increases with the increase of the variance. As a measuring sensor, the strain gauge has certain precision requirements. The result of deterministic analysis *ε* = 9200 με is taken as the exact solution, and the measurement error is considered reliable within 3%. Therefore, it can be concluded that the variance of *α*_4_ should be less than 0.143 × 10^−6^/K to ensure the accuracy and effectiveness of the measurement results.

When the variance of the thermal expansion system *α*_4_ is unchanged and is taken as 0.095 × 10^−6^/K, and the mean value is in the range of [9.0255 × 10^−6^, 9.9755 × 10^−6^], a set of probability density curves of thermal strain *ε* is calculated. The variation of the confidence interval corresponding to the curves with the mean value of *α*_4_ is obtained, as shown in Figure 16. The mean value of *ε* increases with the increase of the mean value of *α*_4_, and the radius does not change with the change of the mean value. To ensure the measurement accuracy of 3%, the mean value of *α*_4_ should be in the range [9.41 × 10^−6^, 9.61 × 10^−6^].

### 4.4. Uncertainty Analysis of Temperature of Grid Wire Based on SFEM

**Figure 17:**
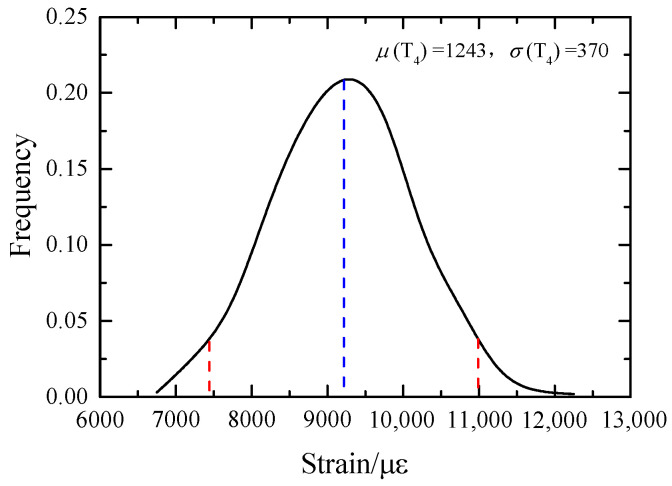
Probability density curve of element 2 thermal strain.

Another major uncertainty source of the system is the grid wire temperature *T*_4_. When *T*_4_ follows the normal distribution (1243.15, 370.15) K, the probability density curve of the output grid wire thermal strain *ε* is shown in Figure 17. It basically follows the normal distribution, with an average value of 9216.4 με and a variance of 886 με. The calculated 95% confidence interval is [7444, 10,989].

**Figure 18:**
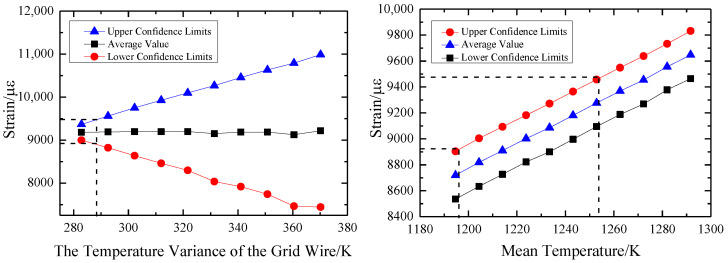
Influence of digital characteristics of *T*_4_ on 95% confidence intervals for thermal strain *ε*.

When the mean value of *T*_4_ is unchanged, its variance is changed. The coefficient of variation *cov* is uniformly selected with 10 values between [0.01, 0.1]. The probability density curve corresponding to the output thermal strain *ε* is calculated, respectively. On this basis, 95% confidence intervals under different variances are calculated, as shown in Figure 18. The confidence interval of output *ε* increases with the increase of *T*_4_ variance, and the mean fluctuation increases with the increase of variance. In the case of a grid wire temperature of 1243.15 K, if the measurement error of the strain gauge is required to be within the range of 3%, the variance of *T*_4_ should be less than 288.15 K.

When the variance of grid wire temperature *T*_4_ is unchanged, it is 282.85 K, and the mean value is uniformly valued in the range of [1194.65, 1291.65]. A group of probability density curves of thermal strain *ε* are calculated, and the confidence interval corresponding to the curve is obtained with the mean value of *T*_4_, as shown in Figure 18. The mean value of *ε* increases with the increase of the mean value of *T*_4_, and the interval radius does not change with the change of the mean value. If the measurement accuracy of 3% is to be guaranteed, the mean value of *T*_4_ should be in the range of [1235.15, 1254.15].

## 5. Conclusions

In this paper, the stochastic finite element method (SFEM), which combines the Monte Carlo method and the finite element method, is used to analyze the load transfer process of the high-temperature strain gauge through a MATLAB program. The calculation results are compared with the thermal–mechanical coupling model of ANSYS. Through these studies, some conclusions can be summarized as follows:

(1) The average temperature of the strain gauge obtained by ANSYS is 1258.44 K. The temperature at the grid wire calculated by MATLAB is 1245.2 K. The difference between the two is 1.34%. The thermal strain calculated by ANSYS at element 2 is 9141 με, which is 0.64% different from the equivalent thermal strain calculated by MATLAB at 9200 με. It is proven that the program is accurate and effective in calculating this complex structure.

(2) The primary sub-model method used in the calculation process can effectively reduce the number of model elements, reduce the requirement of computing resources, and obtain the exact solution of the thermal response of the grid wire.

(3) The SFEM is used to analyze the influence of the uncertainty of 10 inputs (physical parameters, geometric parameters, and load) on the output, thermal stress, and thermal strain of the grid wire. When the coefficient of variation of each parameter is the same value 0.1, the thermal expansion coefficient of the grid wire *α*_4_ and the thermal load *T*_4_ have the most significant influence on the thermal response dispersion. When the *α*_4_ follows the normal distribution (9.5 × 10^−6^, 0.95 × 10^−6^), the mean value of thermal stress *σ_xx_* of the grid wire is -0.6394 Pa, and the variance is 0.06404 Pa. When the *T*_4_ follows the normal distribution (1243.15, 370.15), the mean value of *σ_xx_* is 0.6387 Pa and the variance is 0.06211 Pa.

(4) The influence of the digital characteristics of *α*_4_ and *T*_4_ on the 95% confidence interval of thermal strain *ε* is analyzed. In order to ensure the measurement accuracy of 3%, the mean value of *α*_4_ should be in the range of [9.41 × 10^−6^, 9.61 × 10^−6^], and the variance of *α*_4_ should be less than 0.143 × 10^−6^/K. The mean value of *T*_4_ should be in the range [1235.15, 1254.15], and the variance of *T*_4_ should be less than 288.15 K.

It can be concluded from the above analysis that the MATLAB program can realize the analysis of the load transfer process of high-temperature strain gauges. The method of layered modeling is also applicable to the analysis of the mechanical characteristics of other laminated structures. The primary sub-model can obtain local fine results, and it is also applicable to other situations where the local performance is concerned, such as the situation of a high local stress concentration caused by small holes and grooves. The SFEM combining the MCM and the FFEM is an effective method to analyze stochastic problems. In the future, an uncertainty analysis of the fatigue life of strain gauges will be performed.

## Figures and Tables

**Table 1:** Calculation results of some nodes of the master model.

Nodes	Temperature/K	*x* Coordinates	*y* Coordinates
61	1221.87	0	6
62	1234.52	0	6.1
63	1244.10	0	6.2
64	1253.68	0	6.3
65	1263.26	0	6.4
66	1272.83	0	6.5

**Table 2:** Temperature values of some nodes of the sub-model.

Nodes	Temperature/K	*y* Co-ordinate	*x* Co-ordinate	Nodes	Temperature/K	*y* Co-ordinate	*x* Co-ordinate
7	1240.97	6.16	0	48	1240.97	6.16	0.01
8	1241.88	6.17	0	49	1241.88	6.17	0.01
9	1242.79	6.18	0	50	1242.79	6.18	0.01
10	1243.70	6.19	0	51	1243.70	6.19	0.01
11	1245.20	6.20	0	52	1245.20	6.20	0.01
12	1246.70	6.21	0	53	1246.70	6.21	0.01
13	1247.61	6.22	0	54	1247.61	6.22	0.01
14	1248.53	6.23	0	55	1248.53	6.23	0.01

**Table 3:** Classification of uncertainties.

Uncertainty Factor	Contents Include
Physical parameters	Coefficient of thermal expansion of grid wire *α*_4_, thermal expansion coefficient of the covering layer *α*_3_, elastic modulus of the substrate *E*_3_, thermal expansion coefficient of the transition layer *α*_2_, and elastic modulus of the transition layer *E*_2_
Geometric dimensions	Grid wire diameter *d*_4_, basal thickness *h*_3_, and transition layer thickness *h*_2_
Load	Force load *F* and temperature load *T*_4_

## Data Availability

The data presented in this study are available upon request from the corresponding author.

## References

[1] Ai Y.T., Liu M., Zhang F.L. (2022). Research on Structural optimization Method to improve the life and accuracy of high-temperature strain gauge. Chin. J. Sci. Instr..

[2] Reis M., Castro R., Mello O. (2013). Calibration uncertainty estimation of a strain-gage external balance. Measurement.

[3] Zhu B., Pei H.F., Yang Q. (2023). Probabilistic analysis of wave-induced seabed response based on stochastic finite element method. Rock Soil Mechan..

[4] Khairi N., Rizam M.S., Naimah M., Nooritawati M., Husna Z. (2012). Diameter Stem Changes Detection Sensor Evaluation Using Different Size of Strain Gauge on Dendrobium Stem. Procedia Eng..

[5] Schmid P., Zarfl C., Balogh G., Schmid U. (2014). Gauge Factor of Titanium/Platinum Thin Films up to 350 °C. Procedia Eng..

[6] Kolhapure R., Shinde V., Kamble V. (2017). Geometrical optimization of strain gauge force transducer using GRA method. Measurement.

[7] Liu H., Mao X., Yang Z., Cui J., Jiang S., Zhang W. (2019). High temperature static and dynamic strain response of PdCr thin film strain gauge prepared on Ni-based superalloy. Sens. Actuators A Phys..

[8] Guo Z., Xu J., Chen Y., Guo Z., Yu P., Liu Y., Zhao J. (2019). High-sensitive and stretchable resistive strain gauges: Parametric design and DIW fabrication. Compos. Struct..

[9] Enser H., Kulha P., Sell J.K., Jakoby B., Hilber W., Strauß B., Schatzl-Linder M. (2016). Printed Strain Gauges Embedded in Organic Coatings. Procedia Eng..

[10] Li Y., Wang Z., Xiao C., Zhao Y., Zhu Y., Zhou Z. (2018). Strain Transfer Characteristics of Resistance Strain-Type Transducer Using Elas-tic-Mechanical Shear Lag Theory. Sensors.

[11] Gräbner D., Dumstorff G., Lang W. (2018). Simultaneous Measurement of Strain and Temperature with two Resistive Strain Gauges made from Different Materials. Procedia Manuf..

[12] Larsen M.L., Adhikari S., Arora V. (2021). Analysis of stochastically parameterized prestressed beams and frames. Eng. Struct..

[13] Bartłomiej P., Marcin K. (2023). Numerical convergence and error analysis for the truncated iterative generalized stochastic pertur-bation-based finite element method. Comput. Methods Appl. Mechan. Eng..

[14] Chen C., Dawson C., Valseth E. (2023). Cross-mode stabilized stochastic shallow water systems using stochastic finite element methods. Comput. Methods Appl. Mech. Eng..

[15] Li J., Liu Q., Yue J. (2021). Numerical analysis of fully discrete finite element methods for the stochastic Navier-Stokes equations with multiplicative noise. Appl. Numer. Math..

[16] Ghanem R.G. (2003). Stochastic Finite Elements.

[17] Popescu R., Deodatis G., Nobahar A. (2005). Effects of random heterogeneity of soil properties on bearing capacity. Probabilistic Eng. Mech..

[18] Lagaros N.D., Papadopoulos V. (2006). Optimum design of shell structures with random geometric, material and thickness imperfections. Int. J. Solids Struct..

[19] Palluotto L., Dumont N., Rodrigues P., Gicquel O., Vicquelin R. (2019). Assessment of randomized Quasi-Monte Carlo method efficiency in radiative heat transfer simulations. J. Quant. Spectrosc. Radiat. Transf..

[20] Vadlamani S., Arun C.O. (2020). A stochastic B-spline wavelet on the interval finite element method for beams. Comput. Struct..

[21] Wang B., Cai Y., Li Z., Ding C., Yang T., Cui X. (2020). Stochastic stable node-based smoothed finite element method for uncertainty and reliability analysis of thermo-mechanical problems. Eng. Anal. Bound. Elem..

[22] Do N.-T., Tran T.T. (2023). Random vibration analysis of FGM plates subjected to moving load using a refined stochastic finite element method. Def. Technol..

[23] Talischi C., Paulino G.H., Pereira A., Menezes I.F.M. (2012). PolyMesher: A general-purpose mesh generator for polygonal elements written in Matlab. Struct. Multidiscip. Optim..

[24] Liu Z., Yang W., Wei J. (2014). Analysis of random temperature field for freeway with wide subgrade in cold regions. Cold Reg. Sci. Technol..

[25] Bärnkopf E., Jáger B., Kövesdi B. (2022). Lateral–torsional buckling resistance of corrugated web girders based on deterministic and stochastic nonlinear analysis. Thin-Walled Struct..

[26] Hong C., Yang Q., Sun X., Chen W., Han K. (2021). A theoretical strain transfer model between optical fiber sensors and monitored substrates. Geotext. Geomembranes.

[27] He C., He H., Wang T., Pei J.H. (2018). Modification and prediction of finite element model in thermal environment considering uncertain factors. J. Vibrat. Eng..

[28] Chen J.J., Wang L.G., Li J.P. (2009). Thermal analysis of rod structures with random parameters in steady state random temperature field. Eng. Mechan..

[29] Nastos C., Zarouchas D. (2022). Probabilistic failure analysis of quasi-isotropic CFRP structures utilizing the stochastic finite element and the Karhunen–Loève expansion methods. Compos. Part B Eng..

